# On the Wireless Microwave Sensing of Bacterial Membrane Potential in Microfluidic-Actuated Platforms

**DOI:** 10.3390/s21103420

**Published:** 2021-05-14

**Authors:** Marc Jofre, Lluís Jofre, Luis Jofre-Roca

**Affiliations:** 1 Department of Research and Innovation, Fundació Privada Hospital Asil de Granollers, 08402 Granollers, Spain; 2 Department of Signal Theory and Communications, Universitat Politècnica de Catalunya—BarcelonaTech, 08034 Barcelona, Spain; luis.jofre@upc.edu; 3 Department of Fluid Mechanics, Universitat Politècnica de Catalunya—BarcelonaTech, 08019 Barcelona, Spain; lluis.jofre@upc.edu

**Keywords:** bacteria, elasto-inertial focusing, microfluidics, microwaves, membrane potential, sensing, shear stress, single cell detection, system-on-a-chip

## Abstract

The investigation of the electromagnetic properties of biological particles in microfluidic platforms may enable microwave wireless monitoring and interaction with the functional activity of microorganisms. Of high relevance are the action and membrane potentials as they are some of the most important parameters of living cells. In particular, the complex mechanisms of a cell’s action potential are comparable to the dynamics of bacterial membranes, and consequently focusing on the latter provides a simplified framework for advancing the current techniques and knowledge of general bacterial dynamics. In this work, we provide a theoretical analysis and experimental results on the microwave detection of microorganisms within a microfluidic-based platform for sensing the membrane potential of bacteria. The results further advance the state of microwave bacteria sensing and microfluidic control and their implications for measuring and interacting with cells and their membrane potentials, which is of great importance for developing new biotechnologically engineered systems and solutions.

## 1. Introduction

Recent scientific and technological advancements are enabling a plethora of new interaction and sensing possibilities that may eventually lead to a drastic change in how we connect with cells [1,2]. One important representative case is the convergence of disciplines like micro-, bio-information technologies and cognitive sciences. These technological advances have been crystallized in different brands and concepts known and used in multiple research fields at present, such as Lab-on-a-Chip (LoC) [3] and System-on-a-Chip (SoC). For instance, SoC has sensing, processing and wireless interaction functionalities based on microfluidic and microwave subsystems [4].

**Figure 1:**
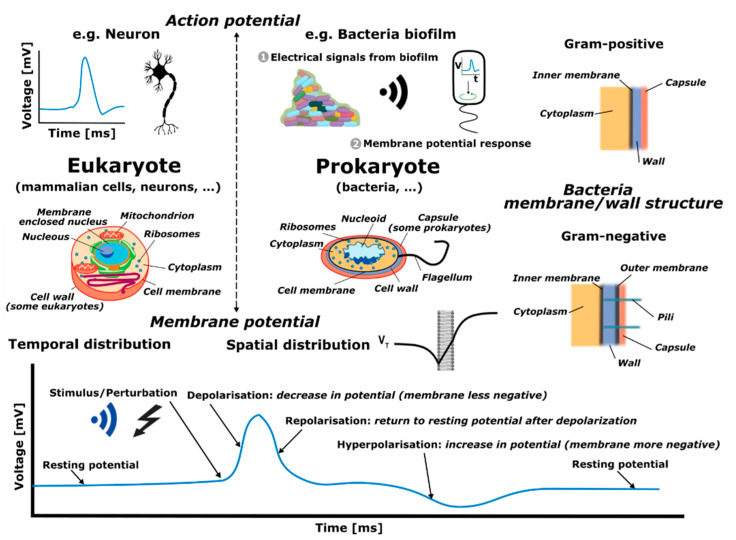
Cells are categorized into eukaryote and prokaryote cells. Additionally, bacteria are categorized according to Gram-negative and Gram-positive, relating to different membrane or wall structure characteristics, as depicted in the upper/middle-right panel. The dynamic process known as an action potential happens in both types of cells. In particular, the membrane potential, given a specific location, changes in time in response to a stimulus or perturbation, as depicted in the bottom graph.

A main area of research in which such disciplines are converging is in biological solutions and technologies involving interaction with cells. A cell is often thought of as the smallest unit of a living organism, made up of even smaller parts, each with its own functionality, as shown in Figure 1. Cells can be categorized into eukaryote (e.g., mammalian cells, neurons) and prokaryote cells (e.g., bacteria). There are several differences between the two, but a major distinction between them is that eukaryotic cells have a distinct nucleus containing genetic material, while prokaryotic cells do not have a distinct nucleus and have free-floating genetic material instead. Besides this distinction, there are several similarities in the different elements that form these two types of cells, such as the cell wall, cell membrane, cytoplasm and ribosomes, among others. Additionally, bacteria can be categorized according to Gram-negative and Gram-positive families (originally due to the permeability of specific dyes when observing them under the microscope), which, as depicted in Figure 1, mostly relates to different membranes or wall structures. Generally, Gram-negative bacteria have an outer membrane, which expresses itself by different responses of the bacteria, for example virulence and antibiotic resistance.

Eukaryote and prokaryote cells, although inherently different as discussed above, present similarities in some functional activities, such as in action and membrane potentials. An action potential is a rapid rise and subsequent fall in voltage that propagates across a cell (such as in neurons), whereas membrane potential refers to the difference in charge between the inside and outside of a cell (such as in bacteria) created due to the unequal distribution of ions on its two sides. Specifically, the term action potential refers to the electrical signaling that occurs within neurons, or bacteria biofilms (aggregation of bacteria), resulting from rapid spatial changes in membrane potential when ion concentrations in the vicinity of membranes abruptly vary. Hence, action and membrane potentials are interconnected since they both depend on the electrophysical differences between the cell and the environment. However, action potentials are transient propagating events, while membrane potentials correspond to pseudo-static electrical characteristics. In this work, we consider sensing tasks as a way of monitoring and interacting with bacteria. In particular, the focus is placed on their membrane potential which, given a specific location, changes in time in response to a stimulus or perturbation, as depicted in the bottom graph of Figure 1. The time response corresponds to an initial, or stable, resting potential (negative voltage value) which then, given a disruption, produces a depolarization (the membrane becomes less negative in voltage), succeeded by a repolarization (the membrane potential returns to the voltage resting potential), followed by a hyperpolarization (increase in negative voltage of the membrane potential), to finally end at the initial/stable resting voltage potential.

The location and time-varying signaling of cells can be described in terms of electromagnetic signals, and consequently research on electromagnetic biosensors is of main importance (an extended discussion is provided in Section 2). Particularly, electromagnetic wave (EM-w) biosensors are attracting a lot of attention due to multiple benefits, such as being minimally invasive and cost effective [5,6,7]. For instance, recent advances in microwave sensing [8,9,10] enable the exploration of new frequency band windows for conducting novel research on membrane interactions. In particular, microwave frequencies allow to efficiently monitor biological elements and properties inside the human body, with considerable penetration distances (in the order of cm) and resolutions (in the order of mm) [11]. When trying to approach this EM-w environment for basic microorganism interaction, the combination of EM biosensors with microfluidics has proven to be a very interesting pairing for testing, interacting and controlling functionality of microorganisms through the interplay with fluid streams [12]; performing these functions without mechanical moving parts, and with little noise generation in the process. In particular, as it will be described in Section 3, inertial and elasto-inertial microfluidics, using Newtonian and non-Newtonian fluids (respectively), can provide efficient single-particle flows in the center of a microfluidic channel and cell focusing that guarantees optimal sensitivity and accuracy [13]. The benefits of such types of combinations will be leveraged in this work to enable the sensing and interaction with membrane potentials.

Therefore, the objectives of this work are to (i) develop a multidisciplinary theoretical framework for designing a microfluidic-based platform to wirelessly detect and interact with the membrane potential of microorganisms, (ii) provide an experimental demonstration of the capabilities that will be enabled with it, and (iii) discuss the results connected with a proposed microwave sensing strategy for the sensing of membrane potentials. In this regard, the paper is organized as follows. First, a description of state-of-the-art EM detection techniques utilized to detect properties of bacteria is provided in Section 2. Next, Section 3 presents the elasto-inertial particle-focusing strategy selected to focus microorganisms at the centerline of microchannels and describes the microfluidic platform designed, while Section 4 focuses on characterizing the requirements for microwave detection of microorganisms. Experimental demonstration of the design and presentation of the results are provided in Section 5. This is followed by Section 6 which discusses the results connected with an original microwave sensing strategy for the detection of membrane potentials. Finally, Section 7 summarizes the work, provides conclusions and proposes future research.

## 2. EM Detection Techniques

**Figure 2:**
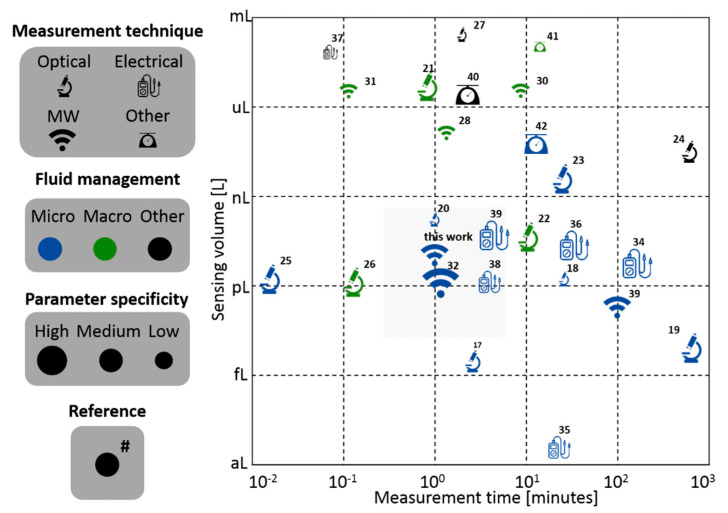
Comparison of several methods for bio-particle detection techniques reported in the literature (optical, microwave, electric and others). In order to compare the different measurement times for different total analyzed volumes, the time was normalized to the equivalent time required to analyze a volume of 100 μL. Fluid management is divided into micro, macro and other. The parameter specificity is divided into high, medium and low.

In many research studies, it is very convenient to detect bacteria individually to monitor and interact with their basic physiological parameters, and in particular with membrane potential on a one-by-one basis. Moreover, individually detecting and analyzing microorganisms is of capital importance in a variety of fields encompassing health, industry and environment technologies, and in applications ranging from diagnosis in clinics and pharma manufacturing to the production of beverages and fuel-treatment of solid deposits, among others [14,15,16]. Figure 2 shows the comparison of several electromagnetic detection techniques reported in the literature based on the measurement time (in minutes) and the sensing volume (in liters). The values provided for the sensing volume correspond to either the geometrical dimension of the sensing volume or the actual liquid volume indicated by the analyzed systems. If multiple values were reported in the same study, the better value (i.e., best performance) is reflected in the chart. The experiments considered were performed with bacteria or particles of similar size to facilitate a better comparison between them on the chart.

The measurement techniques are grouped in four categories: optical, microwave, electric and others. Optical techniques refer to measurement systems where both the microorganism illumination and sensing is performed using light-based techniques [17,18,19,20,21,22,23,24,25,26,27]. Microwave systems make use of electromagnetic illumination and detection at higher frequencies in the order of GHz (>0.1 GHz and <1 THz), where the equivalent wavelength is similar or larger than the fundamental characteristic dimension of the measurement system (this work) [28,29,30,31,32,33]. Electrical techniques consist mainly of approaches that make use of low frequency electrical signals (<0.1 MHz) for measurement, where the equivalent wavelength is much larger than the fundamental characteristic dimension of the measured system and the electromagnetic dielectric properties substantially contribute to the detection process [34,35,36,37,38,39]. Other techniques consist of different physical-based (e.g., mass spectroscopy, magnetic detection) measurement systems that have been proposed or exist for detecting bacteria [40,41,42].

In terms of fluid management, defined as the relevant fluid mechanics principles that govern the system, it is divided into three categories: micro, macro and other. Micro refers to managing fluids in flow channels with cross-sectional dimensions smaller than 100 μm. Macro refers to flows handled in fluidic channels, or containers, with at least one cross-sectional dimension larger than 100 μm. Other fluid-controlled techniques refer mainly to systems in which there is no intentional fluid flow management to make the particles flow through the sensing area.

Finally, the parameter specificity is divided in three levels: high, medium and low. The parameter specificity is defined for measurement techniques that aim to sense a particular physical property of the micro-organisms rather than solely detecting the element as a whole. The high level refers to experiments which have used a labeling technique or the physical measurement extracts high-level information from the bacteria. The medium level refers to studies where the physical measurement extracts qualitative information with little direct relation to the bacteria’s biological state or characteristics. The low level refers to studies that detect bacteria without any further insight. Figure 2 shows a common tradeoff between measurement time and sensing volume. This is expected since (roughly) in order to have more sensibility (smaller volumes sensed) more time is needed to measure a large part of the sample. As seen in Figure 2, there is an important gap in the literature with respect to sensing small volumes and using microwave techniques.

There is no specific technique that reins in all regions but it is a fact that, over the past 30 to 40 years, many advances have been performed in optical [17,18,19,20,21,22,23,24,25,26,27] and low/medium-frequency electrical techniques [34,35,36,37,43]. Differently, this work is focused on microwave techniques at the microfluidic scale, falling therefore in the center of the chart both in terms of sensibility and measurement time, which is of interest for bacteria detection applications, and especially relevant for sensing and interacting with bacterial functional parameters.

## 3. Design of the Microfluidic Platform for Elasto-Inertial Focusing of Particles

**Figure 3:**
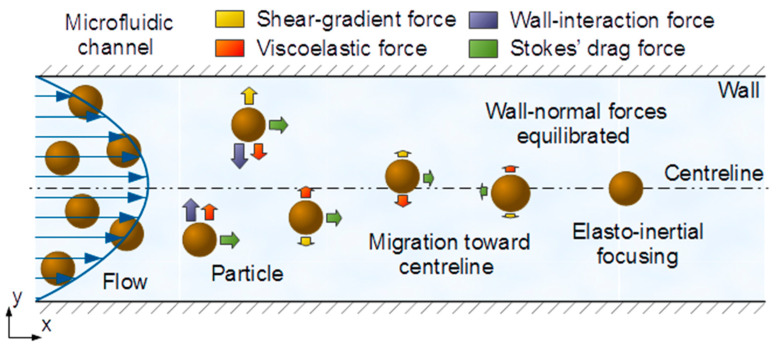
Illustration of the hydrodynamic forces acting on particles in an elasto-inertial microfluidic channel. The particles are randomly distributed at the inlet of the channel. The interaction, and final balance, of the wall-normal forces results in the focusing of the particles at the centerline. The main forces are shear-gradient lift force (yellow), wall-interaction force (violet), viscoelastic force (red) and Stokes’ drag force (green).

Over the past decade, microfluidic technology has been proven essential in biological research to precisely control the motion and position of microorganisms in a fluid flow, and is consequently the methodology selected in this work to complement the microwave-based sensing technique (presented in Section 4) to achieve hydrodynamic focusing of microorganisms. In this regard, inertial focusing is an effective technique for controlling particle positions (particles are utilized as a surrogate of biological species) in microfluidic devices at low-moderate Reynolds numbers (Re), i.e., Re∼1−100; in fluid mechanics [44], Re characterizes the ratio of inertial to viscous forces used to classify flow regimes as laminar (flow organized in layers) or turbulent (flow dominated by velocity fluctuations). As first studied in [45] for Newtonian fluids in a cylindrical pipe, initially randomly distributed particles are known to focus to an annulus located between the center and wall of the pipe, while in square-section channels, following the symmetry of the system, particles instead focus to four equilibrium regions centered at the faces of the flow-bounding walls [46]. However, when utilizing viscoelastic fluids, i.e., a type of non-Newtonian material that exhibits both viscous and elastic behavior when undergoing deformation, particles have been found to migrate toward the centerline of pipes/channels in the case of fluids with constant viscosity [47]. The underlying principle of these phenomena is the balance of the hydrodynamic forces acting on the particles while advected by the flow. Particularly, as illustrated in Figure 3, in the case of elasto-inertial microfluidic systems [18], four main forces need to be considered: (i) wall-interaction force, (ii) shear-gradient lift force, (iii) viscoelastic force and (iv) Stokes’ drag force.

### 3.1. Dimensionless Numbers and Characteristic Regimes

The characterization of elasto-inertial fluid motion and particle focusing in wall-bounded microfluidic flows is efficiently assessed by considering the Reynolds number, defined as $Re_{\;}=\frac{\rho_{f}U_{c}D_{h}}{\mu_{f}}$, where $\rho_{f}\;$ and $\mu_{f}$ are the density and dynamic zero-shear viscosity of the fluid, $U_{c}$ is the centerline fluid velocity, and $D_{h}=2WH/\left(W+H\right)$ is the hydraulic diameter with W and H the width and height of the microfluidic channel, respectively. This is in addition to the Weissenberg number, characterizing the ratio between elastic and viscous forces, given by $Wi=\tau_{f}\dot{\gamma }=\frac{\tau_{f}U_{c}}{D_{h}}$, where $\tau_{f}$ is the fluid relaxation time, and $\dot{\gamma }=2Q/\left(HW2\right)$ is the characteristic shear rate with $Q=WHU_{m}$ the volumetric flow rate and $U_{m}=\left(2/3\right)U_{c}$ the mean fluid velocity in a laminar channel flow. The ratio between these two dimensionless numbers corresponds to the elasticity number $El=Wi/Re_{\;}=\tau_{f}\mu_{f}/\left(\rho_{f}D_{h}^{2}\right)$, which only depends on the channel dimensions and fluid properties. Other parameters that are of second-order importance include the geometry of the channel, the strength of the shear-thinning effect, the initial position of particles and the blockage ratio defined as $\kappa =d/D_{h}$ with d as the particle diameter; when κ<0.25, blocking effects can be assumed to be negligible.

As studied in [48], the equilibrium position for most particles in a viscoelastic fluid is either at $Y_{p}\equiv y_{p}/D_{h}>\approx 0.15$ or at the channel axis, where $Y_{p}$ is the normalized vertical position away from the centerline. This is due to the occurrence of the inertial-force peak at $Y_{p}\approx 0.15$, which is explained below. For a second-order fluid, the viscoelastic force on a particle is $F_{VE}=\frac{-40}{3}\pi \rho_{f}U_{c}^{2}\kappa d^{2}El\;Y_{p}$, with the negative sign indicating that the force drives the particle toward the center of the channel, in contrast to the shear-gradient lift force, which causes the particle to migrate away from the central axis given by [30] $F_{SG}=C_{SG}\rho_{f}U_{c}^{2}\kappa d^{2}$ for $Y_{p}\approx <0.3$, where $C_{SG}$ is the shear-gradient lift coefficient, which is a positive function of $Y_{p}$ and has a maximum value of $C_{SG}\approx 0.05$ at $Y_{p\;}\approx 0.15$, and is equal to zero at both $Y_{p}=0$ and $Y_{p}\approx 0.3$; the wall-interaction force $F_{WI}=-C_{WI}\rho_{f}U_{c}2d^{6}/D_{h}⁴$ ($C_{WI}$ is the wall-interaction lift coefficient) is not considered in this analysis because it is assumed that the particle is close to the centerline and sufficiently away from the wall. The relative magnitude between $F_{VE}$ and $F_{SG}$ determines whether the particle can be focused at the centerline or not. In fluid flows with El≫1, the viscoelastic force overcomes the maximum shear-gradient inertial force, and the particle migrates toward the centerline. In contrast, for fluid flows with El≪1, the particle stops at a location before $F_{SG}$ reaches its maximum. Therefore, the balance between $F_{VE}$ and $F_{SG}$ at $Y_{p}\approx 0.15$ leads to an estimate for the critical elasticity number of $El_{c\;}\approx 0.025∼0.01$.

The channel length L required for particles to achieve equilibrium positions at the centerline, i.e., particle focusing, is based upon the magnitudes of the forces described above and their variation along a particular channel. As an extremely important design parameter, this length must be estimated carefully. Following a scaling analysis for straight channels [13], the focusing length $L_{F}$ can be estimated based on the transversal particle migration velocity $U_{p}$ [49], which was calculated using the balance of $F_{SG}$ and Stokes’ drag force, defined as $F_{D}=3\pi \mu_{f}dU_{p}$, obtaining the expression $L_{F}=\frac{3\pi \mu_{f}D_{h}^{2}}{C_{SG}\rho_{f}U_{c}d^{2}}∼\frac{\pi \mu_{f}D_{h}^{2}}{C_{SG}\rho_{f}U_{c}d^{2}}$, where $C_{SG}$ takes values in the range 0.02–0.05; $F_{VE}$ is zero at the channel centerline and is consequently not considered to estimate $L_{F}$. In the case of significantly small particle Reynolds numbers, i.e., $Re_{p}=Re{\left(\frac{d}{D_{h}}\right)}^{2}\ll 1$, the focusing mechanisms may be degraded due to the diffusion rate of particles becoming comparable to the inertial migration velocities [50]. This limitation is evaluated by means of an equivalent particle Peclet number defined as $Pe_{p}=\frac{U_{p}L_{c}}{D}=\frac{C_{SG}\mu_{f}^{2}D_{h}Re_{p}^{2}}{2k_{B}T\rho_{f}}$, where $L_{c}=D_{h}/2$ is a characteristic length scale, and $D=k_{B}T/3\pi \mu_{f}d$ is the Stokes-Einstein diffusion coefficient of a particle with $k_{B}$ as the Boltzmann constant and T as the temperature of the system. For the diffusion effects to be negligible in inertial microfluidic systems, it is required that $Pe_{p}\gg 1$.

In addition to the inertial and viscoelastic forces creating equilibrium positions for particles within the cross section of a channel, particles suspended in a fluid will interact in a flow with finite inertia to create particle trains with regular spacing in the streamwise direction of the flow. Therefore, particle concentration is also a critical factor affecting the focusing behavior and accuracy. Aside from the potential interparticle interactions, there are wake effects due to particles being concentrated in a few relatively narrow streamlines. In order to identify when these effects become important, the number of particle diameters per channel length, or length fraction, $\lambda_{mf}=d/L$ is defined, which is more appropriate than a volume fraction given that particles are focused to single streams. One can convert from a volume fraction α to $\lambda_{mf}$ using the relation $\lambda_{mf}=\frac{6HW\alpha }{\pi d2}$. For $\lambda_{mf}\gg 1$, focusing to a single stream is not to be expected due to wake interactions between particles, whereas the opposite is true for $\lambda_{mf}\ll 1$. Note that for a given α, $\lambda_{mf}$ increases quadratically with decreasing d, such that accurate focusing of smaller particles requires significantly more (quadratically) diluted solutions.

### 3.2. Microfluidic Design for Microwave-Based Sensing of Bacteria

**Figure 4:**
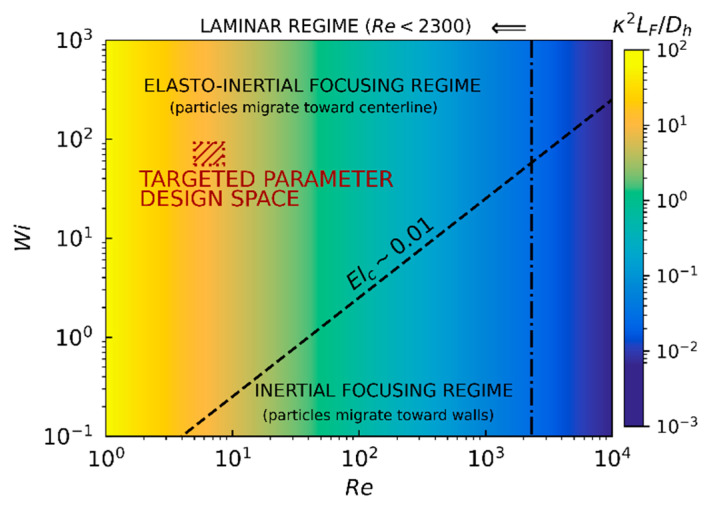
Regime diagram of the dimensionless Reynolds (Re) and Weissenberg (Wi) numbers for viscoelastic fluids of inertial flows in microfluidic channels. The colormap corresponds to the squared blockage ratio (κ) multiplied by the slenderness ratio ($L_{F}/D_{h}$); a value of $C_{SG}=0.05$ has been utilized. The diagonal dashed line (critical elasticity number $El_{c}∼0.01$) separates elasto-inertial from inertial focusing regimes, while the vertical dot-dashed line separates laminar (Re<2300) from transitional/turbulent flow regimes. The red dashed rectangle indicates the parameter design space targeted for sensing bacteria using microwave-based techniques.

Elasto-inertial microfluidics can be prepared by additives comprising biological, or synthetic, polymeric powders. For example, 500 ppm of polyethylene oxide (PEO) polymer in a phosphate-buffered saline (PBS) solution can be used to force particles to focus into a single stream at the center of a microfluidic channel [17]. As explained above, elasto-inertial focusing is achieved under specific conditions which depend mainly on the Reynolds and Weissenberg numbers and, to a second-order approximation, on the blockage ratio and length fraction. The interplay between these different parameters is visually summarized on the regime diagram depicted in Figure 4. The vertical critical-Reynolds-number ($Re_{c}\approx 2300$) line separates the laminar flow region, where the balance between forces discussed in this section applies, from the transitional/turbulent region not typically achieved in microfluidic applications due to their small characteristic sizes and velocities. In parallel, the diagonal critical-elasticity-number ($El_{c}∼0.01$) line delineates the separation between elasto-inertial and inertial-focusing regimes. The red dashed rectangle indicates the parameter design space, which is detailed below, targeted for sensing bacteria using microwave-based detection techniques.

The constraints of the designed microfluidic channel are imposed by manufacturing, sensing, fluid properties and biological limitations, and correspond to: (i) the width, height and length of the channel are W=H=50 μm and L=60 mm, as standardized by manufactured microchips; (ii) the average fluid velocity provided by the pressure pump is tuned to $U_{m}=0.2\;m/s$ (the range $U_{m}=\left[0.17-0.225\right]\;m/s$ is considered to calculate the targeted parameter design space) to provide sufficient exposure time (approximately 250 μs) for sensing the biological species on a field of view of 50 μm as typically required by a microscope; (iii) the viscoelastic fluid utilized is based on a solution of water with 500 ppm of 0.4 MDa PEO solution and resulting in $\rho_{f}=1000\;\mathrm{kg}/m^{3}$, $\mu_{f}=1.95\;\mathrm{mPa}\cdot s$ and $\tau_{f}=11.39\;\mathrm{ms}$ [18]; and (iv) the diameter of the bacteria considered is d∼1 μm, with a reasonable particle number density for preparing solutions in the order of $n_{p}∼5\times 10^{4}\;\mathrm{particles}/\mathrm{mL}$. This set of values results in the elasto-inertial microfluidic design located within the red dashed rectangle depicted in Figure 4 and corresponding to Re≈8, which is smaller than 2300 (laminar regime), Wi≈68 which is larger than 1 (viscoelastic fluid), El≈9 which is larger than $El_{c}∼0.01$ (elasto-inertial focusing regime), $Pe_{c}\approx 11$ which is sufficiently larger than 1 (inertial regime), κ=0.02 which is smaller than 0.25 (blocking effects are negligible), Q=30 μL/min, $\lambda_{mf}∼1.25\times 10^{-4}$ which is much smaller than 1 (dilute solution), and $L_{F}∼1\;m$ (a value of $C_{SG}=0.05$ has been utilized) which is more than an order of magnitude larger than L=60 mm.

The inadequacy of the design in terms of focusing length, i.e., $L_{F}∼1\gg L=0.06\;m$, is expected due to the small diameter of the particles considered; elasto-inertial manipulation of particles is typically limited to $Re_{p}\gg 0.1$ as $L_{F}$ scales inversely with $d^{2}$. A solution recently proposed to overcome this challenge is based on oscillatory microfluidics [50]. Unlike traditional steady-flow microfluidics, oscillatory microfluidics switches the streamwise direction of the flow at a specific frequency. Due to the symmetry of the velocity field along the flow axis, the elastic and inertial forces acting on the particles preserve their wall-normal directionality when the flow is switched, and consequently, by exploiting this symmetry, the focusing length can be extended indefinitely, even though the channel itself has a much shorter fixed length. Based on the dimensionless numbers described above, the frequency of the oscillations $f_{mf}$ and the corresponding distances traveled by the particles can be defined for a given system. In particular, $f_{mf}$ is limited in the lower end by the distance L that the particles can travel within the microchip. On the higher end, $f_{mf}$ is limited by the entrance length, viz. streamwise distance required for the flow to develop. In the case of laminar flow, this upper limit is quantified by the dimensionless Womersley number, which expresses the pulsatile flow frequency in relation to viscous effects, defined as $Wo=D_{h}\sqrt{\frac{2\pi f_{mf}\rho_{f}}{\mu_{f}}}$. Small Womersley numbers, i.e., Wo<1, indicate that the flow is fully developed for most of the oscillation period, and consequently entrance-length effects can be ignored. In general, the wall-normal particle focusing positions achieved by this oscillatory flow method are the same as for traditional steady-flow systems [51]. Particularly for the microfluidic design considered in this work, selecting an oscillation frequency based on the mean velocity of the fluid and a length equal to the channel length, i.e., $f_{mf}=U_{m}/L$, the Womersley number obtained is Wo=0.16<1, which is sufficiently small for entrance-length effects to not impact the elasto-inertial focusing of particles.

## 4. Design of the Microwave Detection Sensor

**Figure 5:**
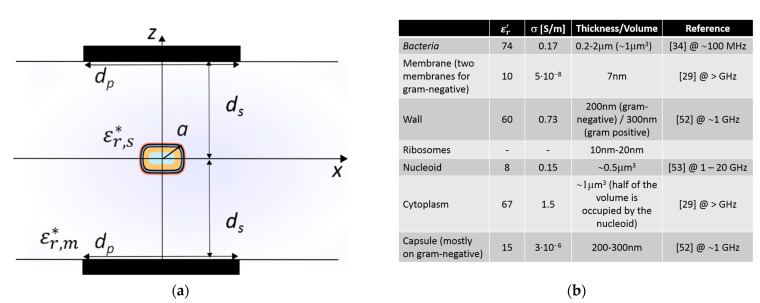
The electromagnetic response of bacteria depends on their shape and size, their internal structure and the complex relative permittivity of the different bacterial cell components, which may depend on the bacterial physiological state. (**a**) Scheme of the configuration and relevant values considered, where $\varepsilon_{r,s}^{*}$ stands for complex relative permittivity of the single cell, a radius, $\varepsilon_{r,m}^{*}$ complex relative permittivity of the surrounding medium, $d_{p}\;$ is the length of the illumination and detection plates, and $d_{s}$ is the distance to the cell (considered equally spaced). (**b**) Real relative permittivity, conductivity and thickness/volume of the different parts of bacteria have been extracted from [52,53] and are congruent with [29,34].

In specific scenarios (like in the problem of interest here), electromagnetic waves interact with particles of material composition differently than the surrounding environment, and the corresponding electromagnetic-matter interaction process is usually referred to as scattering. In this work, we will center the analysis on this phenomenon to monitor biological particle functional dynamics. A general scheme of the configuration of electromagnetic illumination and detection of scattering by a small particle (e.g., bacteria or polystyrene beads) in a liquid medium (e.g., water) is depicted in Figure 5. One of the goals in scattering experiments is to infer information about the complex relative permittivity of the particle from measurements of the scattered field. Here, particle refers to a region in space characterized by a dielectric complex permittivity $\varepsilon_{s}^{*}$, or equivalently a dielectric complex relative permittivity $\varepsilon_{r,s}^{*}$ of the single particle. $\varepsilon_{r,s}^{*}=\varepsilon_{r,s}^{\prime }-j\varepsilon_{r,s}^{\prime \prime }=\varepsilon_{r,s}^{\prime }-j\frac{\sigma_{s}}{\omega \varepsilon_{0}}$, where $\varepsilon_{r,s}^{\prime }$ is the real relative permittivity, $\varepsilon_{r,s}^{\prime \prime }$ is the relative loss factor, $\varepsilon_{0}$ is the dielectric constant of the vacuum and $\sigma_{s}$ is the dielectric conductivity, which is different from that of the surrounding medium (indicated with subscript m) $\varepsilon_{r,m}^{*}=\varepsilon_{r,m}^{\prime }-j\frac{\sigma_{m}}{\omega \varepsilon_{0}}$. In particular, at microwave frequencies, and for the typical dimensions of the experimental setup, the conditions of scattering are of near-field (in contrast to higher frequencies where the conditions are of far-field). Furthermore, since the particle can be assumed to be isolated due to the dilute solutions required for hydrodynamic particle focusing (significantly smaller particle-fluid volume fractions), a single scattering event is considered.

### 4.1. Scattering by a Small Spherical Particle

When an electromagnetic wave impinges on a microorganism, the incident wave can be modeled as a uniform electromagnetic wave (this is sufficiently accurate since the emitter is much larger than the particle) in the case of assuming: (i) the electromagnetic wave to be polarized in the z direction, and (ii) the particle to be a small spherical region of radius a with complex permittivity $\varepsilon_{s}^{*}$. Essentially, the particle sees a constant field as the plane wave impinges on it (viz. an almost electrostatic field on the incident field). The incident field polarizes the particle, making it resemble an electric dipole. Since the incident field is time-harmonic, the small electric dipole will oscillate and radiate like a Hertzian dipole in the far field. However, considering the near field of the particle, a Hertzian dipole can be efficiently approximated by a small current source of value $\vec{J}\left(\vec{r}\right)=\hat{z}Il\delta \left(\vec{r}\right)$, with a corresponding vector potential $\vec{A}\left(\vec{r}\right)=\frac{\mu }{4\pi }∭d\vec{r^{\prime }}\;\frac{\vec{J}\left(\vec{r^{\prime }}\right)}{\left|\vec{r}-\vec{r^{\prime }}\right|}e^{-j\beta \left|r-r^{\prime }\right|}=\hat{z}\;\frac{\mu Il}{4\pi r}e^{-j\beta r}$, where μ is the permeability, I is the time harmonic current, l is the length of the dipole, r is the distance from the particle and β is the complex wavenumber.

#### Near-Field Regime

In the near-field regime, the electric field is given by $\vec{E}=-j\omega \vec{A}-\nabla \Phi$, where the scalar potential term dominates over the vector potential of the scatterer. The scalar potential $\vec{\Phi }\left(\vec{r}\right)$ is obtained from the Lorenz gauge as $\nabla \cdot \vec{A}=-j\omega \mu \varepsilon^{*}\Phi$, and results in $\Phi \left(\vec{r}\right)=\frac{-1}{j\omega \mu \varepsilon_{s}^{*}}\nabla \cdot \vec{A}=\frac{-Il}{j\omega \varepsilon_{s}^{*}4\pi }\frac{\partial }{\partial z}\frac{1}{r}e^{-j\beta r}$. When close to the dipole, by assuming that βr≪1, a quasi-static approximation can be utilized for the potential (equivalent to ignoring retardation effects) $\frac{\partial }{\partial z}\frac{1}{r}e^{-j\beta r}\approx \frac{-1}{r^{2}}$, and reducing the potential to $\Phi \left(\vec{r}\right)\approx \frac{ql}{4\pi \varepsilon_{s}^{*}r^{2}}$. The dipole induced by the small particle is formed in response to the incident field. The incident field can be approximated by a constant local static electric field ${\vec{E}}_{inc\;}=\hat{z}\;E_{i}$. The corresponding electrostatic potential for the incident field is then $\Phi_{inc}=-\hat{z}\;E_{i}$, so that ${\vec{E}}_{inc}\approx -\nabla \Phi_{inc}=\hat{z}E_{i}$. The scattered dipole potential from the spherical particle in the vicinity and in the same axis of illumination is given by $\Phi_{sca}=E_{s}\frac{a^{3}}{r^{2}}$. The electrostatic boundary problem is $E_{s}=K_{CM}E_{i}$, where $K_{CM}=\frac{\varepsilon_{s}^{*}-\varepsilon_{m}^{*}}{\varepsilon_{s}^{*}+2\varepsilon_{m}^{*}}$ is the Clausius-Mossotti factor. As a result, the electric scattered near-field is given by ${\vec{E}}_{sca}=\left[\frac{k^{2}a^{3}}{r}-\frac{a^{3}}{r^{3}}\right]K_{CM}E_{i}\;\hat{z}$. Moreover, the associated electric field power is $P_{E}={\left|{\vec{E}}_{sca}\right|}^{2}/\eta_{w}$, where $\eta_{w}\approx 60\lambda /r$ is the near-field electrical wave impedance of the medium for a wavelength λ [54]. Furthermore, in terms of electrical circuit equivalent representation of the scattered fields, as a bio-particle flows through the sensing electrodes, the perturbed electrical field (scattering) results in a capacitance change between the electrodes [10]. This change can be derived from the perturbed system energy caused by an induced dipole moment $\vec{p}$ as $\Delta U=\frac{1}{2}\Delta CV_{i}^{2}=-\frac{1}{2}\vec{p}\cdot \vec{E_{i}}$, where $V_{0}$ is the driving voltage. Hence, the induced capacitance change can be expressed as $\Delta C=4\pi a^{3}ℜ\left\{\varepsilon_{m}^{*}K_{CM}\right\}\frac{{\left|E_{i}\right|}^{2}}{V_{i}^{2}}$.

### 4.2. Microwave Scattering of Bio-Particles

In microwave standards [55,56], $100\;W/m^{2}$ ($10\;\mathrm{mW}/{\mathrm{cm}}^{2}$) is a typical magnitude of radiation fluxes below which thermal effects are typically negligible. For instance, in this work, the calculations are estimated assuming an irradiance of around $80\;W/m^{2}$ ($8\;\mathrm{mW}/{\mathrm{cm}}^{2}$) at a distance of 25 μm from the particle and with a 50 μm×50 μm effective field of view. Considering the above irradiance and field of view, the power intensity is $P_{i}=200\;\mathrm{nW}$ applied on a 50 Ω resistor (for common impedance matching for RF instrumentation) in parallel with the high equivalent resistance of the electrodes. Furthermore, the equivalent E-field, assuming the electrodes being parallel plates, is $E_{i}=\frac{V_{i}}{2d_{s}}$, where $V_{i}$ is the applied voltage between the electrodes and $2d_{s}$ is the separation between the electrodes.

**Figure 6:**
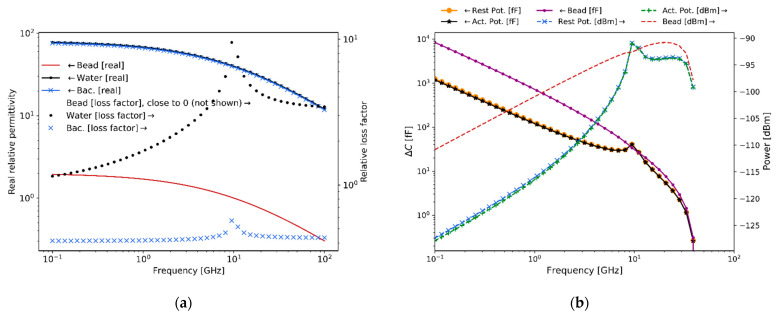
Microwave complex relative permittivity, expected capacitance change and scattering signal levels (near-field) for small 1 μm particles. (**a**) Complex relative permittivity values for the frequency range 0.1−100 GHz  approximated from [29,34,52,53], with addition of (i) water, (ii) bacteria in an action potential process and (iii) polystyrene beads. (**b**) Expected capacitance change and scattering detection signal levels.

Generally, in sensing experiments, the complex relative permittivity of polystyrene beads differs substantially from the complex relative permittivity of the surrounding medium (water), resulting in a better scattering detectability. However, the complex relative permittivity of cells at rest, or when generating a potential, does not differ much from the medium. As a result, cells are almost transparent at microwave frequencies compared to the medium in which they are submerged; a similar scenario is encountered at optical frequencies. The membrane’s capacitance (related to the real permittivity) of bacteria depends on its surface area, while the dielectric loss factor of the membrane depends on its cross-sectional area [57]. Furthermore, at microwave frequencies, in contrast to frequencies <10 KHz, the bacteria’s dielectric values of complex relative permittivity does not change significantly for different states of a cell [58,59], as shown in Figure 6a. Hence, at microwave frequencies it is not expected to easily observe a large variation between a cell at rest and one generating a potential because these physical dimensions are not expected to change significantly. Moreover, the expected capacitance change and scattering signal for 1 μm polystyrene bead and bacteria are depicted in Figure 6b, where an ample 5% [58,59] complex relative permittivity change at microwave frequencies has been assumed. However, in practice at microwave frequencies no complex relative permittivity change between the bacteria in a potential state and in a rest state should be expected since the α and β dielectric polarization dispersions are not present [5]. Hence, Figure 6b shows the challenge at microwave frequencies of achieving detection discrimination of bacteria at a potential state with respect to bacteria at rest. Furthermore, in Figure 6a, the peak at 10 GHz of the relative loss factor of water and bacteria corresponds to the γ dispersion relaxation phenomenon due to intermolecular hydrogen bonding [5]. Lastly, the required sensitivity for detection is shown in Figure 6b. These estimations are crucial to assess the required performance of the measuring instrumentation utilized for obtaining the experimental results presented in Section 5.

The above expected capacitance change, or equivalent scattering detection signal levels, were calculated considering $80\;W/m^{2}$ irradiance intensity. As mentioned before, such irradiance intensity ($<100\;W/m^{2})$ at microwave frequencies is considered to not produce thermal-related effects. Such types of effects need to be considered when the temperature is increased more than 1 K in tissues or bio-particles [60]. Estimating the amount of microwave heating generated requires the consideration of energy conversion from electromagnetic to thermal energy. In detail, microwave energy is delivered directly to the bio-particles through molecular interaction with the electromagnetic field, often known as dielectric heating. Dielectric heating refers to heating by the electric field (E-field) component of the high-frequency electromagnetic spectrum. This is due to the presence of electric dipoles in polar molecules, which are sensitive to external electric fields and will attempt to align themselves with the field through rotation. Under a high frequency electric field (e.g., >10 GHz), the dipoles do not have sufficient time to respond to the oscillating field, and as a result of this phase lag, they collide with each other when they attempt to follow the field. The result is that power is dissipated and the material consequently heats up. This phenomenon is known as the dipolar polarization mechanism and is the basis of microwave dielectric heating [61]. The heat produced can be quantified from the average power dissipated $P_{av}=\frac{1}{2}\omega \varepsilon_{r,s}^{\prime \prime }\varepsilon_{0}{\left|E_{i}\right|}^{2}$ as described in [5], where *ω* is the angular frequency, $\varepsilon_{r,s}^{\prime \prime }$ is relative loss factor of the cell, $\varepsilon_{0}$ is the dielectric constant of the vacuum, and $E_{i}$ is the incident field. Utilizing the expression for the average power dissipation, the associate temperature change in time can be calculated as $\frac{\Delta T}{t}=\frac{P_{av}}{m_{p}C_{p}}$, where $m_{p}=\rho_{p}V_{p}$ is the mass and $C_{p}=1308\;J/\left(\mathrm{kg}\cdot K\right)$ is the specific heat capacity at constant pressure of the bio-particle (e.g., bacteria) [62]; $\rho_{p}=1050\;\mathrm{kg}/m^{3}$ and $V_{p}=\left(4/3\right)\pi {\left(2a\right)}^{3}$, with radius a=0.5 μm are the density and volume of the particle. As the change in temperature depends on the time duration of the irradiance intensity, the determination of whether heat effects have to be considered or not strongly depends on this later quantity. In particular, for illumination times similar to the membrane potential duration (10 ms−100 ms), the calculated temperature increase is below 0.3 K for the frequencies of interest in this work (0.1−100 GHz). Furthermore, for frequencies below the 10 GHz resonance frequency, the temperature increase is much smaller due to less relaxation effects that reduce the number of inter-collisions. As a result, for the problem of interest in this work, the increase in temperature produced by illumination will not be important, and consequently heat effects do not need to be considered in the calculations. However, in the case of larger illumination times (seconds and above), these thermal effects would need to be taken into account, especially for frequencies above 10 GHz.

## 5. Experimental Results

**Figure 7:**
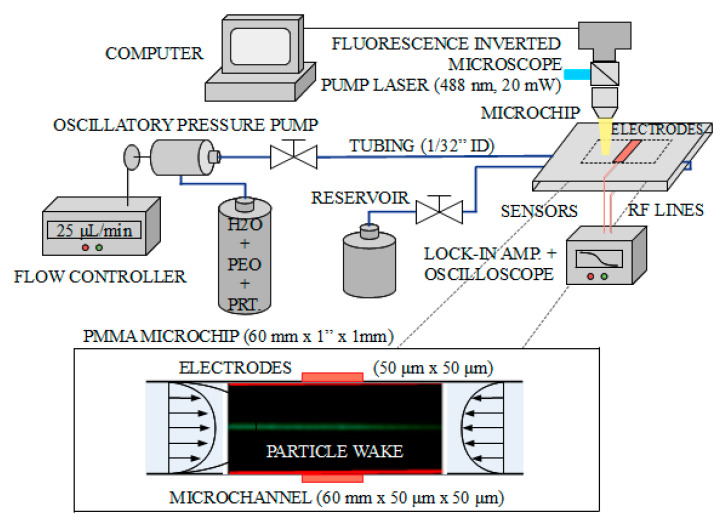
Schematics of the experimental setup. The system is computer controlled and consists of an inverted fluorescence microscope with image sensor, a pressure-driven flow-controlled microfluidic pump, and an electromagnetic measurement station composed of a lock-in amplifier and a visualization oscilloscope. The microfluidic chips used have connectorized square cross-section channels with cross-sections of 50 μm and 100 μm, and with a length of 60 mm. The electrodes are integrated within the microchips and connected through RF lines to the lock-in amplifier.

As described in Section 3, to achieve single-cell detection (also for membrane potential sensing, as proposed in Section 6) elasto-inertial hydrodynamic focusing combined with oscillatory-fluid operation is utilized in this work to (i) position a single bio-particle at the centerline of the microchannel (equidistantly from the measuring electrodes located at the microchannel walls), and (ii) to allow sufficient time to sense/measure the biological parameters of interest. In this regard, Figure 7 provides a schematic illustration of the experimental setup combining the microfluidic platform, electromagnetic measurement platform and microscope for visualization and measurement purposes. The system is computer controlled and consists of an inverted fluorescence microscope (different microscope objectives available ranging from 10× to 100×) with an integrated color image sensor (CMOS camera with 1 ms frame rate with external triggering, 5 mLux sensitivity and 100 μs minimum integration time) and equipped with a manually controlled micro-positioning stage with a custom 488 nm 20 mW pump laser. The microscope fluorescence cube consists of a band-pass 460∼490 nm excitation filter, a 500 nm dichroic mirror and a long-pass 520 nm emission filter. On the microfluidics side, the fluid is driven by means of a pressure-driven flow-controlled pump, capable of providing 30 psi of maximum pressure. The different microfluidic components are connected using tubing of 1/32” of internal diameter, combining polyetheretherketone (PEEK) and polytetrafluoroethylene (PTFE) tubing across the microfluidic system. The internal diameter (ID) is constant throughout the system to reduce the quantity of bubbles generated and enhance the stability of the flow. The oscillatory fluid operation is achieved using standard slow-switching three-way valves. The electromagnetic measurement station is connected using 50 Ω-matched coaxial cables from the microfluidic chip to a lock-in amplifier, and visualized in a low bandwidth time oscilloscope. All the electromagnetic parts are properly shielded and grounded to avoid and reduce possible electromagnetic interferences present in the laboratory.

The microfluidic channels available for the measurements correspond to commercially available components with optical-grade-quality polymethyl methacrylate (PMMA) and two different cross-sections (50 μm and 100 μm), are 60 mm long, and contain integrated 50 μm×50 μm electrodes. Figure 7 (bottom) shows an example of the microfluidic measurement provided by the microscope system. The focusing of particles (polystyrene fluorescent beads) was demonstrated for 10 μm (mean size of 10.2 μm with range 10–14 μm) and 1 μm (mean size of 1 μm with range 0.8–2 μm). The different samples were prepared in 10 mL of volume using 0.2 μm-filtered water, more than four-weeks-cured 500 ppm PEO from 10,000 ppm of 0.4 MDa prepared stock, and with $10^{5}\;\mathrm{beads}/\mathrm{mL}$. The procedure to operate the experiment is the same for the two channel cross-sections. First, the pressure controller (0∼30 psi) transmits pressurized air (positive pressure pump, with maximum of 116 psi and adjusted to 30 psi) to the first reservoir containing the sample preparation and this initiates the circulation of the fluid. The fluid then passes through a debubbler (porous PTFE with 0.5 μm pores, 97 μL internal volume), whose function is to remove any air bubbles that are contained within the fluid. It then passes through the flow sensor (1 μL/min accuracy and 80 μL/min maximum flow rate), and enters the microchip oscillating at 30 Hz, to complete the cycle by finally ending in the waste reservoir.

**Figure 8:**
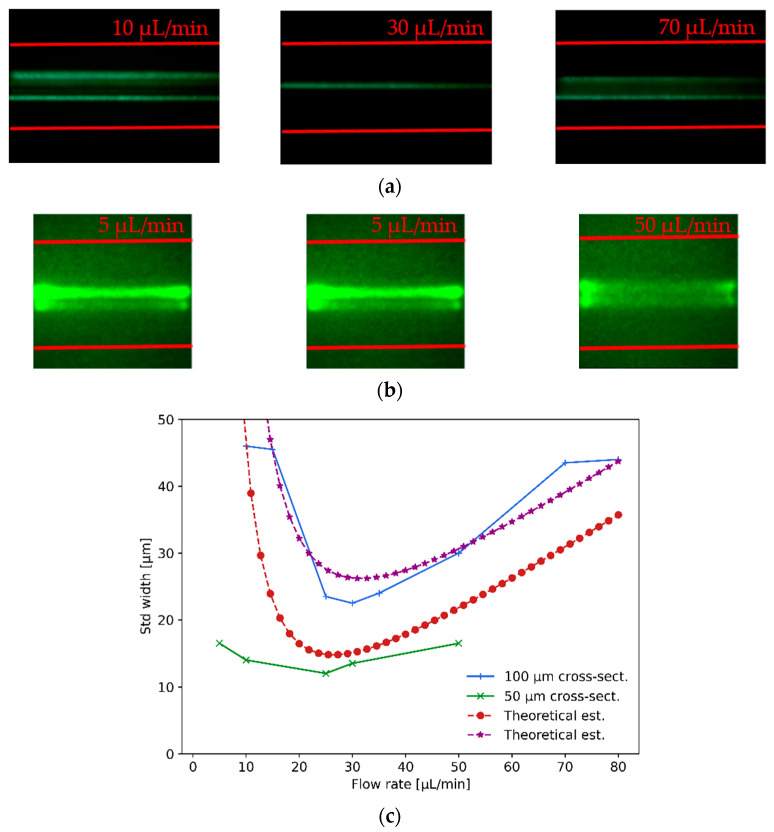
This figure shows 10 μm and 1 μm particle visco-elastic hydrodynamic focusing measurements: (**a**) 10 μm particle hydrodynamic focusing (optimum focusing for ∼30 μL/min) was assessed in a 100 μm cross-section channel, imaged with a 20× microscope objective; (**b**) 1 μm particle hydrodynamic focusing (optimum focusing for 25 μL/min) was assessed in a 50 μm cross-section channel, imaged with a 40× microscope objective. (**c**) For the two different beads sizes, the quality of the hydrodynamic focusing was assessed in terms of the standard deviation width of the particle wake measured and compared to theoretical estimations.

As shown in Figure 8a, the first test consisted of hydrodynamically focusing 10 μm-size polystyrene fluorescent beads in the 100 μm cross-section microfluidic channel by varying the flow rate from 10 μL/min to 80 μL/min. In Figure 8a, the utmost left image shows different beads passing through the channel (particle wake) at relatively off-centered distances when the flow rate is below the optimal focusing value (the red lines indicate the microchannel walls). Idem for the utmost right image where the flow rate is above the optimal one. On the contrary, the center image in Figure 8a depicts the beads passing mostly through the centerline of the microfluidic channel with a high degree of hydrodynamic focusing (achieved for ∼30 μL/min flow rate). From left to right, the images for the 10 μm beads in the 100 μm cross-section channel correspond to flow rates of 10 μL/min, 30 μL/min and 70 μL/min, respectively. Similarly, the hydrodynamic focusing of 1 μm-size beads was assessed, shown in Figure 8b, in the 50 μm cross-section microchannel. Once again, when the flow rate was too small or too large, optimal focusing was not achieved, while for 25 μL/min the optimal flow rate provided an efficient hydrodynamic focusing of particles. The images from left to right for the 1 μm beads in the 50 μm cross-section channel correspond to flow rates of 5 μL/min, 25 μL/min and 50 μL/min. The images were captured with 20× and 40× microscope objectives in fluorescence configuration, respectively, and were processed with standard image processing techniques to compute the standard deviation width of their recorded wakes.

In Figure 8c, it is shown that the measured values (green crosses and blue plus signs) of hydrodynamic particle focusing agree with the results obtained from the theoretical estimations (red dotted and purple star curves) described below. An estimation of the lateral migration velocity $U_{p}$ can be derived (first-order approximation) in the vicinity of the channel centerline by balancing the shear-gradient inertial lift force with the Stokes drag force as $U_{p}∼C_{sg}\rho_{f}U_{m}^{2}d^{3}/3\pi \mu_{f}D_{H}^{2}$. Noticing that the Stokes-Einstein diffusion coefficient of the particle is $D=k_{B}T/3\pi \mu_{f}d$, the transversal standard deviation width is then limited to $L_{T}∼D/U_{p}$; in the plot, the estimation has been corrected to account for high-order non-linear effects at relatively large flow rates (>30 μL/min). As shown in Figure 8c, the expected standard deviation width obtained from the theoretical estimation is 20 μm at a flow rate of 25 μL/min, whilst the experimental measurements fall just above and below these expected values with standard deviation values of 25 μm and 12 μm for the 50 μm and 100 μm cross-section channels, respectively.

**Figure 9:**
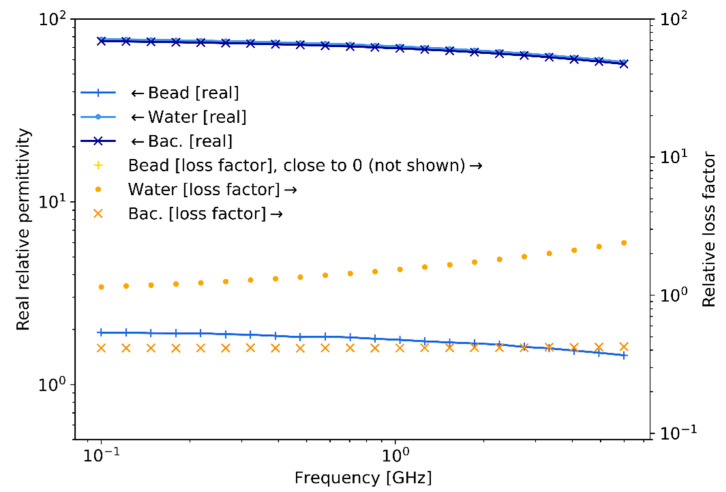
Microwave complex relative permittivity for different media. Measured complex relative permittivity of water, beads and bacteria in the range 0.1–6 GHz using the techniques described in [63,64], which are in concordance with [29].

Figure 9 summarizes the measured experimental values of bacteria complex relative permittivity in the frequency range 0.1–6 GHz using the technique described in [63,64]. A vector network analyzer (VNA) with two built-in ports, with 91 dB of dynamic range, 2 MHz–6 GHz of frequency range, and −4 dBm of output power, is employed to measure the S-parameters, which are post-processed and transformed to complex relative permittivity. These results, although measured for bacteria in a culture sample, are still indicative of the trends that would be observed for single-bacteria measurements and are consequently useful for estimating the expected measurable capacitance change and scattering levels for the latter case.

As expected, the real relative permittivity for water and bacteria are of a similar value of around 80, while they are approximately 2 for the polystyrene beads. These values remain relatively constant across the frequency range. On the contrary, the relative loss factor for water and bacteria presents a uniform difference of a half-decade for almost all frequencies. In addition, the value of the relative loss factor for polystyrene beads (not shown) remains close to 0 throughout the frequency range.

The electromagnetic measurement is centered in a lock-in amplifier, which generates the reference 100 MHz signal and is directly applied to the microfluidic chip through the available pads of the DC-high-impedance electrodes, as indicated in Figure 7. This signal is also utilized internally for the phase-lock receiver to detect small voltage signals embedded in the noise. The lock-in amplifier is 50 Ω-matched, with a single-ended input channel, working with voltage signals, a $5\;\mathrm{nV}/\sqrt{\mathrm{Hz}}$ noise level and maximum working frequency of 200 MHz. The applied 100 MHz signal on the electrodes is adjusted to 3.2 mV on a 50 Ω-matched circuit prior to being transferred to the electrodes; this approach is equivalent to applying an irradiance of around $80\;W/m^{2}$ through the micro-channel. While the impedance of the micro-channel at DC frequencies is equivalent to an open circuit (the resistance between the electrodes is in the order of giga-ohms), it reduces to hundreds of ohms at the MHz–GHz range due to the equivalent parallel capacitance produced by the microchannel. The time integration filter of the lock-in amplifier is set to 100 μs, which corresponds to the time-of-flight duration of the particles within the effective field of view of the electrodes for each pass of the oscillatory flow regime. The interference noise above 10 KHz is fairly well reduced by the lock-in amplifier, while the 50 Hz electric grid noise (Europe) is properly shielded and filtered out by enclosing the open electromagnetic circuits in a metallic-box structure (with anti-reflection foams in the inner face to avoid electromagnetic self-resonances within the enclosure).

**Figure 10:**
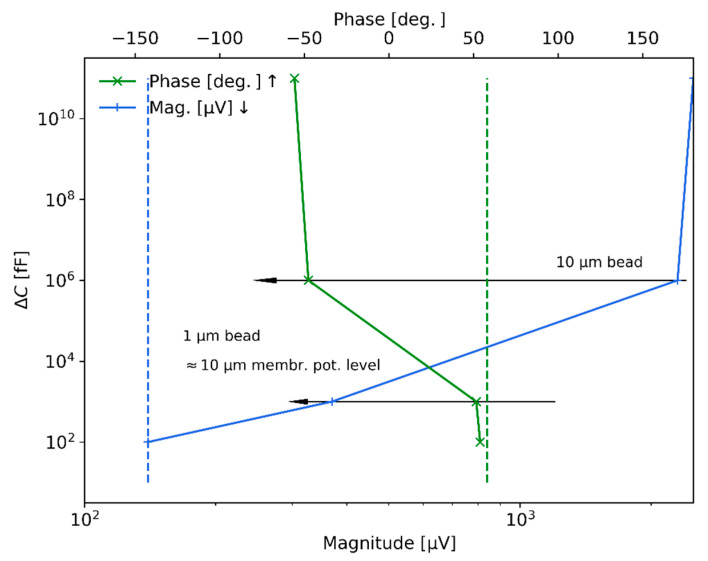
Electromagnetic measurement results for 1 μm and 10 μm polystyrene beads transformed into equivalent capacitance change. The blue and green solid lines represent the detected levels of voltage magnitude and phase, while the dashed lines represent the sensitivity limit of the system (magnitude and phase).

Figure 10 shows the measured results with the lock-in amplifier when measuring 1 μm and 10 μm polystyrene beads in the 50 μm×50 μm cross-section chip with integrated electrodes separated by 50 μm (approximately 25 μm of separation from the top/bottom electrode to the particles) at a flow rate of 25 μL/min. The bottom horizontal axis represents the magnitude signal in V of the detected peaks when the beads pass through the sensing zone of the microchannel, while the top horizontal axis indicates the phase signal change detected. In particular, the measured values were in the order of 2 mV (around −50 deg. phase value) for the 10 μm bead, while they significantly reduced to 300−400 μV (around 54 deg.) for the 1 μm beads; as a reference, for the case of the microchannel full of air and without particles, the detected magnitude was 2.5 mV (around −58 deg). The signal sensitivity level achieved with the electromagnetic measurement system is around 150 μV in magnitude and 58 deg. in phase (shown in Figure 10 by dashed lines for the voltage magnitude and the phase). Regarding the vertical axis of the figure and considering the theoretical descriptions provided in Section 4, the levels of signal detected are transformed to equivalent capacitance change units. In particular, the transformed capacitance changes for the 10 μm beads are around nano-farads, while the equivalent capacitance change level for 1 μm beads is found in the range of pico-farads. Hence, the sensitivity achieved with the current measurement system is around hundreds of femto-farads.

The levels of hydrodynamic focusing achieved and electromagnetic capacitance changes (or sensitivity) measured from the experimental rig and discussed in this section are of main importance for demonstrating the capability of the designed system to sense the biological signals of bacteria and more generally of other potential microorganisms, for example neurons and their action potential. Moreover, as proposed in this work and presented in the following section, it is also important to notice that being able to measure the signal produced by a 1 μm bead shows, as will be discussed in the next Section, that the system could be used to measure the second-order non-linear membrane potential response of 10 μm bio-particles since both signals are expected to be of the same order of magnitude.

## 6. Discussion on Membrane Potential Detection

Cellular, as well as intracellular, membranes exhibit a distinct nonlinear electrical behavior due to the potential barrier resulting from the difference between the inner and outer electrolytes and the action of ion-pumps [65]. In the absence of an applied electromagnetic field, the transmembrane potential difference $\Delta_{\phi }$ is equal to the cell resting potential $V_{0}$ (≈−100 mV for a typical cell). When a cell enters into an active membrane potential state it generates itself a low frequency voltage transmembrane voltage excess potential $\Delta_{\phi }=V_{0}+\delta_{\phi }$. On top of the self-generated low frequency voltage, the illuminating signal at microwave frequencies (the cell is relatively sensitive to this electromagnetic frequencies) adds a high frequency small-signal transmembrane voltage excess potential on the cell, which is proportional to the electric field between the electrodes. As a result, a transmembrane current density ${\vec{J}}_{m}$ is generated. The current-voltage response is known to be fairly well approximated by a nonlinear diode-like relationship of the form $J_{m}=J_{0}\left(e^{\delta_{\phi /V_{T}}}-1\right)$ with typical values of order $J_{0}\approx 10^{-5}\;A/{\mathrm{cm}}^{2}$ and $V_{T}\approx 5\;\mathrm{mV}$ [66,67].

In terms of electromagnetic flux, the current density associated with the membrane potential generated by bacteria is typically in the order of $10\;\mu A/{\mu m}^{2}$. This relatively high, but extraordinarily localized in space (membrane of bacteria) and time (10–100 ms), density current could generate two-way coupling phenomena between the electromagnetic signal and fluid flow as analyzed in [68]; in their work, the fluid studied is dilute in electrical charges, and consequently the electromagnetic forces are finally neglected. Similarly in this problem, the impact of the electromagnetic signal on the fluid phase, which is also electrically dilute, in addition to be activated sporadically and for a small period of time, exponentially decays as $1/r^{2}$ (diffusion term in Poisson-Nernst-Planck-Stokes equation in spherical coordinates [69]). As a result, the net effect will not be powerful enough to substantially modify the balance of hydrodynamic forces governing the advection and focusing of bacteria in the centerline of the microchannel.

**Figure 11:**
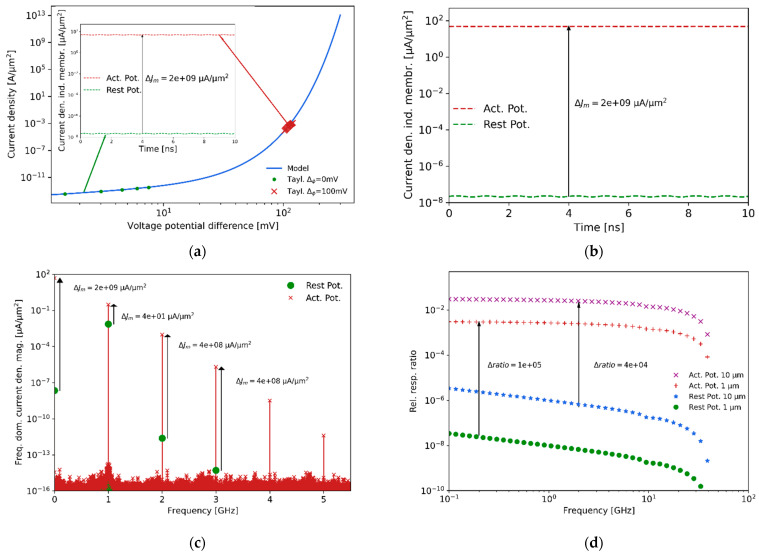
Nonlinear voltage-current response of the membrane potential. (**a**) Current density non-linear dependence with the differential voltage potential. (**b**) Induced current density due to the self-generated transmembrane potential and the impinging microwave field at two different membrane potential states, while (i) at rest and (ii) generating an active potential. (**c**) Frequency domain analysis where the fundamental frequency scattering is due to the membrane. (**d**) Frequency plot of the expected relative second-order responses at rest and at an action potential state.

In general, at optical frequencies the use of a generated second-order signal to sense the potential variation of cells has been demonstrated by making use of the nonlinear susceptibility tensor [70,71]. In this work, we proposed to sense the membrane’s action potential at microwave frequencies by leveraging the non-linear behavior of the voltage-current relationship of the membrane potential. Ideally, the charges respond to the rapid microwave wavelength, as indicated in [72]. As shown in Figure 11, the cell’s membrane is illuminated with a microwave field (e.g., 1 GHz), while the membrane’s voltage working point in the non-linear voltage-current model will be determined by the cell’s self-potential state, as shown in Figure 11a. Depending on the state of the membrane potential, the scattered signals will have multiple generated frequency harmonics (due to the non-linear voltage-current model of the membrane) in comparison to the incident illuminating signal. In particular, using a Taylor series expansion for the first two terms of the transmembrane current density $J_{m}$, the expected efficiency of the second-order process at $\delta_{\phi }=100\;\mathrm{mV},$ compared to $\delta_{\phi }=0\;\mathrm{mV},$ is $4\times 10^{8}$ times larger, while the second-order process at $\delta_{\phi }=100\;\mathrm{mV}$ compared to the first-order one is $2.5\times 10^{-3}$, which is still significantly efficient [73].

In addition, Figure 11b, which is the insight plot of Figure 11a, shows the induced current density due to the self-generated transmembrane potential and the impinging field at two different membrane potential states, while (i) at rest and (ii) generating an active potential. Figure 11c shows the resulting frequency harmonics generated due to the nonlinear voltage-current response of the membrane potential. The fundamental frequency scattering intensity due to the membrane thickness (around 10 nm) is obscured compared to the scattering generated by the cell’s bulk body (difference ratio of intensities around 4), making it very difficult to sense the difference between the bacteria at rest and in an active potential state. Instead, the second-order, for instance, is characterized by a difference of approximately 8 orders of magnitude between the two states. The reason for this result is that the frequency mixing involved in the transmembrane voltage excess potential is directly sensing the effect of the membrane potential and not the entire body of the bacteria. The expected relative second-order response at different illumination frequencies, with respect to the fundamental frequency at rest and at an action potential state, are shown in Figure 11d. As a note, the size of the bacteria scales the response with the cube of the radius, and consequently it is more challenging to detect 1 μm than 10 μm cells.

**Figure 12:**
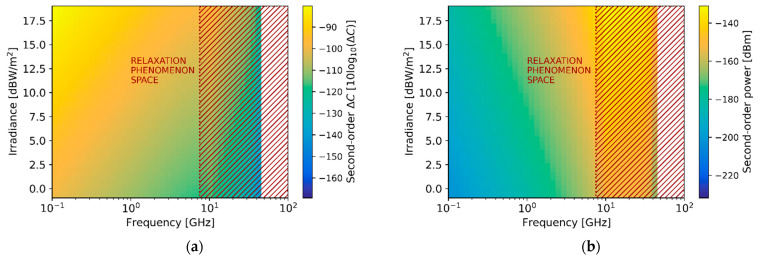
Second-order generated signal power dependence with illumination irradiance and frequency for 1 μm bio-particles. (**a**) Colormap plots of the capacitance change (**a**) and scattering level (**b**) associated with the second-order response with respect to the irradiance and frequency.

Figure 12 shows the expected magnitude of the second-order signal with respect to the illumination frequency in the 0.1–100 GHz range and the irradiance power applied in the tens of $\mathrm{dBW}/m^{2}$ range. In particular, Figure 12a depicts the response in units of capacitance change, while Figure 12b presents it in units of scattering signal level. The figure, as it is common for frequency conversion, shows the non-linear relation between irradiance intensity and power of the generated harmonic. Moreover, the second-order non-linear response vanishes strongly above 10 GHz due to the low efficiency of the processes caused by the relaxation phenomena (red-dashed rectangles).

**Figure 13:**
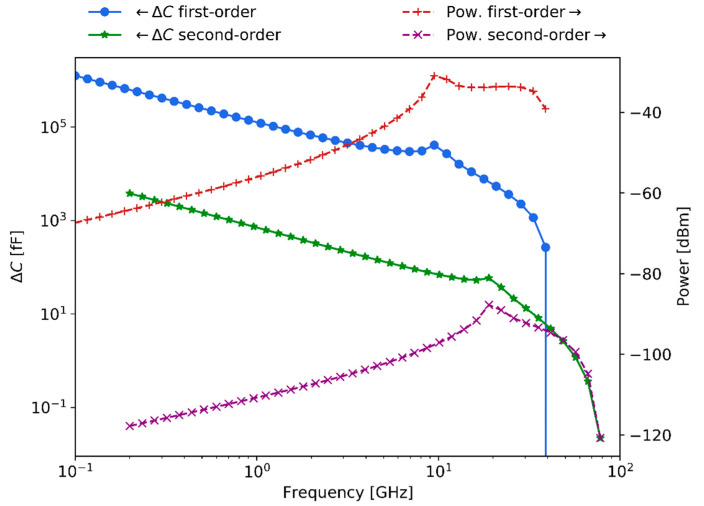
The signal levels regarding capacitance change and scattering levels for first-order (detection) and second-order (sensing of membrane potential) for 10 μm-sized bio-particles.

Finally, Figure 13 presents the first-order expected capacitance change and scattering levels of detection when considering an irradiance power of $80\;W/m^{2}$ and the illumination geometry chosen in this work. In addition, the figure also depicts the second-order response, which in this work is proposed to be related to the sensing of the membrane potential.

The capacitance change of the first-order curve is in the 1∼1000 pF range, while the second-order response is in the 1∼1000 fF range. Equivalently, the scattering signal level of the first-order is in the −70∼−40 dBm power range, whereas the second-order response is in the −120∼−90 dBm power range. As expected, the capacitance change diminishes with increasing frequencies while scattering signals increase. Hence, the calculations indicate that at large frequencies scattering detection techniques are convenient since larger signal responses are expected to be generated. Relevantly, the expected membrane potential sensitivity levels for 10 μm bio-particles fall within the experimentally demonstrated sensitivity results for the 1 μm polystyrene beads, presented in Section 5.

## 7. Conclusions

Recent observations of the signaling roles of the membrane potential of bacteria have drawn the attention of researchers to study the dynamics of microorganisms, many of which remain unexplored particularly at microwave frequencies. Furthermore, elasto-inertial microfluidics can provide efficient hydrodynamically focused single-particle flows convenient for particle handling and sensing. Therefore, acquiring microwave interaction knowledge of bacterial membrane potential dynamics in microfluidic-based platforms is essential to advance our understanding of how to apply these models.

In this work, we have summarized the general concepts of bacteria’s membrane potentials. Furthermore, we have revised several electromagnetic detection techniques for bacteria together with a comparison between most of the relevant features among them. A microfluidic framework suited for such investigations was defined, quantified and assessed with laboratory measurements. In addition, we discussed the detection of bacteria using scattering microwave techniques, and measurements of the complex permittivity of bacteria and sensitivities for polystyrene 1 μm and 10 μm beads were presented for the analysis. Finally, we proposed a method for enhancing and particularizing the detection of the membrane potential dynamics in the microwave range. The expected sensitivity for sensing the membrane potential of 10 μm bio-particles with the proposed method was supported with the experimental results presented.

Fortunately, the requirements of microfluidics and microwave-based techniques converge to the need of miniaturization for sensitivity. However, microwave-microfluidic sensing and interaction with cells is still a largely uncharted territory in microbiology. We look to further apply this combined approach that holds the promise of facilitating new scientific discoveries in the areas of cell action and membrane potentials and will ultimately help advance the status of controlling and sensing technologies.

## Data Availability

Not applicable.
